# A New Vision at the Interface of Atrial Fibrillation and Stroke

**DOI:** 10.3389/fcvm.2021.689313

**Published:** 2021-08-09

**Authors:** Rafael M. Ronsoni, Marco Aurélio Lumertz Saffi, Marcus Vinicius Magno Gonçalves, Igor Hidetsu Nakayama, Tiago Luiz Luz Leiria

**Affiliations:** ^1^Electrophysiology Department, Instituto de Ritmologia Cardíaca, Joinville, Brazil; ^2^Department of Medicine, Universidade da Região de Joinville, Joinville, Brazil; ^3^Department of Cardiology, Hospital de Clínicas de Porto Alegre, Porto Alegre, Brazil; ^4^Programa de Pós-Graduação em Ciências da Saúde - Instituto de Cardiologia do Rio Grande do Sul/Fundação Universitária de Cardiologia, Porto Alegre, Brazil

**Keywords:** atrial fibrillation, stroke, atrial myopathy, cardioembolic stroke, review

## Abstract

**Introduction:** Current evidence questions the linear sequence traditionally described in atrial fibrillation, blood stasis, intracavitary thrombus, and embolization to the central nervous system. Currently, new perspectives have been described based on questions from the linearly traditional chronology of events; it is within this scope that the article has its objective.

**Evidences:** The association of the two entities is biologically plausible and supported by different cohorts with a higher risk of developing atrial fibrillation, especially in the cardioembolic form. Concepts (temporal dissociation, biological gradient, etc.) determine the existence of other factors associated with cardioembolism, not exclusively by atrial fibrillation. The entire cascade of events associated with myopathy and atrial remodeling can generate damage to the myocyte and amplify the prothrombotic status. It is important to clarify that atrial myopathy can present itself as atrial fibrillation initially or not, but should always be considered thrombogenic in all the contexts of their clinical presentation. Considering atrial heart disease as a cause of embolic stroke, it could explain that one-third of strokes are considered cryptogenic.

**Conclusions:** The traditional model exclusively associating the presence of atrial fibrillation in the genesis of thromboembolism is incomplete. The concept of atrial cardiopathy where cardioembolism occurs in a non-atrial fibrillation dependent manner fits better with current data. The future challenge is to effectively detect the various manifestations of atrial heart disease, generating direct implications for the identification of patients at risk of stroke and also for better management after a cardioembolic event.

## Introduction

Atrial fibrillation (AF) is considered the most common sustained arrhythmia in clinical practice and with complex pathophysiology, occurring in normal hearts even in the presence of the most varied structural diseases (1–3). Initial results of studies from the Framingham cohort (4) determined the relationship with morbidity and mortality, especially its relationship with stroke (5, 6). Its prevalence has reached epidemic proportions, resulting in a perspective that one in four American or European adults will have AF diagnosed in the near future, without considering a rate between 10 and 25% of asymptomatic cases (2, 3, 7, 8).

Current evidence questions the linear sequence traditionally described in AF, blood stasis, intracavitary thrombus, and embolization to the central nervous system. Currently, new perspectives have been described based on questions from the linearly traditional chronology of events; it is within this scope that the article has its objective (9).

## Evidence Between AF and Stroke

### Positive Points in the Relationship

The association of the two entities is biologically plausible and supported by different cohorts with a higher risk of developing AF (three to five times), especially in the cardioembolic form (10–12). The association of arrhythmia with the most diverse cardiovascular risk factors associated with stroke is true (age, male gender, diabetes, high blood pressure, heart failure, among others), but despite the possible confounding effect, the association remains independently associated after statistical control of the factors risk (10, 11). And what further strengthens the relationship is that AF remains associated with clinical and neuroimaging stroke patterns related to cardioembolism (13).

### Interrogations in the Relationship

However, some doubts are raised, one of which is the association of AF with non-cardioembolic stroke; for example 10% of patients with lacunar stroke have AF and the neurological event associated with the atherosclerosis of large vessels is twice as common in patients with AF. Therefore, the risk of stroke in the context of AF cannot be fully explained by the direct association of AF and stroke, through the cardioembolism pathway, that is, it can occur by other mechanisms (12).

If AF is directly responsible for cardioembolism, it is difficult to understand why elderly patients with cardiovascular risk factors with subclinical AF are associated with twice the risk of stroke as opposed to younger patients with isolated AF who do not have a significant risk of stroke in clinical follow-up. Therefore, these data question the clear biological gradient between stroke and AF; this suggests the occurrence of other associated mechanisms in the elderly population (14, 15).

Another important concept is the temporal dissociation between arrhythmic events and AF. Up to one-third of patients with paroxysmal AF do not show arrhythmic episodes prior to neurological ictus and some will only present an arrhythmic event long after the stroke (16–18). This concept determines the existence of other factors associated with cardioembolism, not exclusively by AF. Another fact that contributes to this question is that the strategies of rhythm control adopted in the treatment of AF were not able to reduce stroke in the clinical follow-up of these patients, despite the apparent success. This has even modified the main trials of rhythm control for AF that have come to value outcomes such as mortality and reduced heart failure, which apparently currently have a stronger relationship with the maintenance of cardiac rhythm (19).

A possible confounding effect is that strokes that affect autonomic centers play a role in triggering AF after neurological stroke. This situation may be a confusing effect in the etiological investigation of stroke, and AF may be unduly responsible for the genesis of the neurological event (20, 21).

## Cardioembolic Stroke

### Epidemiology of Cardio-Embolic Stroke

Cardioembolic stroke is responsible for about 15–20% of all ischemic events. In developed countries the trend is toward a higher prevalence in relation mainly to atherothrombosis, reaching up to 34.7% (22–25). This is closely linked to better control of atherosclerosis risk factors, such as hypertension and dyslipidemia, generating greater relevance in countries such as Canada. Cardioembolic stroke has tripled its incidence over the past few decades and has a prospect of a further 3× increase by the 2050s in developed countries (26).

Atrial fibrillation is a well-known risk factor for ischemic stroke, causing a five-fold increase in stroke risk, reporting a prevalence of AF in patients with ischemic stroke of about 25–30%. This prevalence is generally underestimated since up to 3 months of follow-up there is an addition of around 20% of new AF diagnoses in previously sinus patients (24).

Atrial fibrillation was most frequently associated with infarcts of the total anterior circulation, occurrence of multiple and bilateral vascular territories accounting for a worse outcome in terms of mortality at 30 days and 1 year, and rate of stroke recurrences in the first year of follow-up (24, 27).

Furthermore, we cannot overlook the possible role of AF in strokes classified as cryptogenic, whose prevalence reaches around one-third of ischemic events. This is explained by the presence of AF in a paroxysmal form that can go undiagnosed during the etiological investigation of the event. Another important fact is that prolonged cardiac monitoring (≥30 days) adds statistically to the diagnoses of subclinical AF in stroke patients initially classified as cryptogenic (28, 29).

### Risk Factors for Cardioembolic Stroke

#### Atrial Fibrillation

The mechanisms associated with AF and stroke have already been highlighted. It is closely linked to stroke, increasing its chance by three to five times in the arrhythmia sufferer. Over the next few decades, the number of patients with AF will double and the estimate of arrhythmia-related stroke will triple (26).

#### Systolic Heart Failure

The main mechanisms associated with intracavitary thrombi are: regional stasis, hypercoagulable state and the association with undiagnosed subclinical AF. This triples the risk of stroke in this population (30).

#### Patent Foramen Ovale

The supposed mechanism is associated with paradoxical embolism through the venous to arterial system through FOP. The evidence is contradictory in the causal relationship between the two clinical entities, except in stroke patients under 50 years of age (28).

#### Aortic Arch Atheroma

The presence of enlarged, ulcerated, non-calcified, or movable plaques have been associated with stroke and these characteristics can affect up to 8% of the population over 45 years of age (31). A common problem is that transesophageal echocardiography is not a method used in all services, which ends up underestimating its real prevalence. Currently, the recurrence rate is around 3%, reflecting the better management of atherosclerosis in secondary prevention nowadays (32).

#### Heart Valve Prosthesis

In the current era of anticoagulation, the risk of stroke in patients with mitral valve is 1.3 and 0.8% in the aortic position, with a lower rate in biological prosthesis than in mechanical ones (33).

#### Other Causes

Infectious endocarditis (IE); approximately one in every 5 IE has CNS embolization, generating a 20× greater risk of stroke within 30 days of disease progression (26).

## New Concept of Atrial Myopathy and Its Relationship With Stroke

### Definition of Atrial Myopathy

Atrial myopathy is considered as any structural, architectural, contractile, or electrophysiological change that affects the atria with the potential to produce relevant clinical manifestations (34).

Pathologically, a series of clinical situations (ventricular and valve cardiomyopathies, sleep apnea syndrome, arterial hypertension, diabetes mellitus, obesity, among others) are associated with structural and electrical changes, which are called “atrial remodeling.” Many conditions can coexist and accelerate the process, but the presence of AF greatly stimulates remodeling (35).

**Figure 1:**
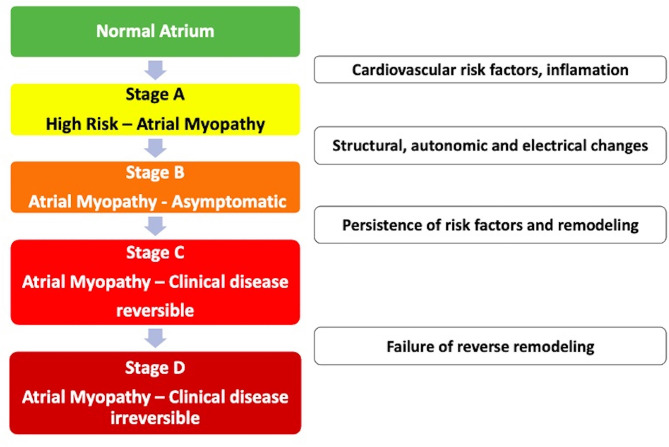
Evolutionary stages of atrial myopathy.

It is important to differentiate three fundamental concepts when studying atrial changes; in some cases, they can coexist:

- Atrial remodeling: response of atrial myocytes to electrical, mechanical, or metabolic stressors, such as rapid atrial tachyarrhythmia, volume or pressure overload leading to persistent changes in size, function and electrophysiological properties of the left atrium (35);
- Atrial myopathy: *Zipes* first described it in 1997. This is the clinical situation that occurs when myocardial diseases are associated with mechanical or electrical dysfunction that usually develop with atrial fibrosis, hypertrophy, or dilation and occur for a variety of causes. Cardiovascular risk factors, such as the clinical factors of the *CHA2DS2-VASC* score, can cause atrial myopathy even in the absence of AF. Example: isolated atrial fibrosis leading to the worsening of atrial function and the development of symptoms of heart failure and atrial arrhythmias (Figure 1) (35, 36);
- Atrial insufficiency: Any atrial dysfunction causing worsening of cardiac function and symptoms of worsening quality of life or life expectancy, in the absence of significant valve or ventricular diseases. Example: high density of atrial fibrosis causing stroke with isolated AF in a zero-point *CHA2DS2-VASC* score. Another example: advanced atrial dyssynchrony causing abnormal ventricular filling and symptoms (35). In summary, external aggressions to the atrium (structural heart disease, hypertension, and diabetes) associated or not with the presence of AF induce a slow and progressive remodeling process. It is important to note that structural remodeling, in general, occurs prior to the arrhythmic clinical presentation, and some components are irreversible. Therefore, early treatment of clinical conditions associated with AF is recommended, even before the electrical manifestation in tachyarrhythmia (8, 37). There are already some electrocardiographic risk markers, such as supraventricular ectopies, atrial tachycardia, and left atrial overload associated with stroke regardless of the diagnosis of AF. These markers of atrial dysfunction are specifically associated with cryptogenic and/or embolic stroke and not with the occlusion of small cerebral vessels (38–44).

### Histological and Pathophysiological Classification of Atrial Myopathy

The world's leading cardiac arrhythmia societies have published the only available classification on myopathy, called by the acronym *EHRA*. It is important to note that it is a classification that is not related to severity or temporal progression (Table 1) (34).

**Table 1:** Classification on myopathy, called by the acronym EHRA.

**EHRAs**	**Histology**	**Clinical correlation**
I	Hypertrophy and myocytolysis of cardiomyocytes. Absence of fibrosis or interstitial changes	Isolated AF, genetic disease, diabetes mellitus
II	Predominance of fibrosis (collagen deposition). Normal cardiomyocytes	Elderly and smoking
III	Mixture of classifications I and II	Heart failure and valvulopathies
IV	Alteration of the interstitial matrix with no collagen accumulation. Accumulation examples: amyloid, fat, inflammatory cells, among others	Amyloidosis, inflammatory diseases, granulomatosis, among others

**Figure 2:**
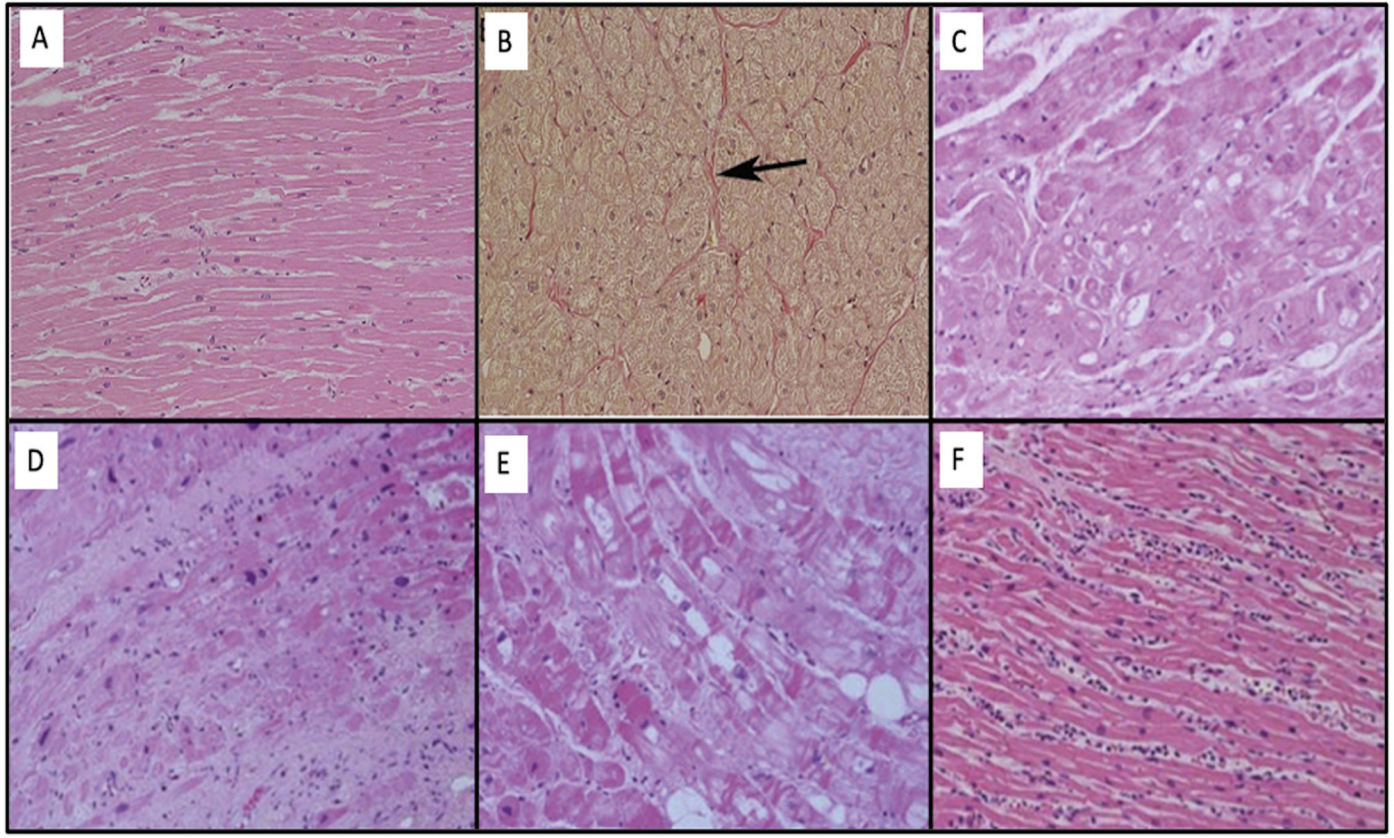
**(A)** Normal histology of the left atrium with the presence of extensive bands of homogeneous myocytes. **(B)** Even the atrium in Panel **(A)** with Van Gieson stain demonstrates that collagen fibers (red color) are identified in the adventitious space of blood vessels (arrow). **(C)** EHRAs Class I: modifications mainly associated with the myocyte through hypertrophy and myocytolysis. **(D)** EHRAs Class II: modifications mainly associated with fibrotic changes. **(E)** EHRAs Class III: mixture of changes in myocyte changes and fibrosis. **(F)** EHRAs Class IV: neutrophilic myocarditis.

The normal characteristics of atrial myocytes are normal below. They have some characteristics of their own such as a prominent sarcoplasmic reticulum (Z tubules) and granules adjacent to the Golgi apparatus that determine natriuretic peptides (e.g. BNP). The others are similar to ventricular myocytes (nucleus, contractile apparatus, cytoskeleton, and organelles). The cellular interstitium is divided into a cellular component (fibroblasts, myofibroblasts, adipocytes, mesenchymal, and inflammatory cells) and extracellular, being mainly of type I collagen fibers. Importantly, collagen is a normal and essential component for the myocytes, being responsible for 5% of normal atrial wall volume. The atrial myocardium is the site of insertion of the post-ganglionic nerve endings of the so-called intrinsic cardiac nervous system, especially in the areas of fat pads. Histological changes to the EHRA classification are shown in Figure 2 (34).

### Atrial Remodeling

The maintenance of AF occurs through a series of structural and electrical changes, which are called “atrial remodeling.” A number of clinical situations external to the atrium are associated with slow and progressive remodeling: ventricular and valvular cardiomyopathies, sleep apnea syndrome, arterial hypertension, diabetes mellitus, and obesity, among others. Many conditions can coexist and accelerate the process, but the presence of atrial fibrillation greatly boosts remodeling (35).

The structural basis for the emergence of AF lies in a remodeling process, with proliferation and differentiation of fibroblasts into myofibroblasts; cell hypertrophy, in addition to extracellular matrix deposition, necrosis, and fibrosis; increased cytoplasmic vacuolization, loss of myofibrils, glycogen accumulation, alteration in mitochondrial size, sarcoplasmic reticulum fragmentation, nuclear chromatin dispersion, and connexin alterations, especially connexins 40 and 43 (36, 45).

In association, there are also amyloid, fatty, and myocardial inflammatory infiltration and, in some cases, metabolism alteration, as seen in atrial ischemia due to coronary disease (36, 46). This disorganization of the atrial tissue generates dilation, greater compliance, and lesser contractility, creating an anatomical substrate for the emergence and perpetuation of multiple reentrant and anisotropy circuits, data confirmed in autopsies (36). In the presence of AF, there is an accumulation of intracellular calcium, resulting in adaptive and inflammatory responses, generating apoptosis and, consequently, atrial fibrosis (36).

Inflammation is closely linked to AF, as suggested by higher concentrations of inflammatory markers identified in the persistent form than paroxysmal in sinus rhythm. Advancing age is considered a risk factor for cardiovascular diseases, such as AF, as it causes a decline in cardiac structure and function; the mechanisms are not fully elucidated. Inflammatory mediators are also associated in elderly people with AF, which may be a possible explanation for the correlation of both entities. Inflammatory markers already identified in high concentrations are C-reactive protein; interleukin types 1B, 2, 6, and 8; fibrinogen; and tumor necrosis factor (36).

Activation of the renin-angiotensin-aldosterone system is also implicated in the amplification of atrial remodeling by several pathways, increasing the susceptibility to atrial fibrillation (46). The system promotes extracellular matrix fibrosis, leading to atrial repolarization heterogeneities and predisposition to the development of AF. Angiotensin II can increase the activity of cardiomyocytes in the pulmonary veins, which can trigger AF (2).

After the onset of arrhythmia, the electrical and mechanical disorganization imposed on the atria intensifies the atrial remodeling process, leading to the perpetuation of the arrhythmia (47).

The studies of Wijffels et al. (48) and Morillo et al. (49) identified a significant decrease and dispersion of the atrial refractory period and a loss or inversion of the adaptation property of the refractory period in relation to the stimulation frequency in experimental models of AF. These changes are likely in the ionic environment and are mediated mainly by slow Ca (L-Ca^2^) channels and electrical remodeling affects calcium and potassium channels (Ito e Ikur) (36, 50, 51).

Both studies demonstrated an increased susceptibility to the induction and sustainability of AF in relation to the time of induced tachycardia, proving that AF leads to AF (“*AF begets AF*”) (48, 49). Another mechanism involved was the decrease in wavelength; the induction of multiple wave-fronts allowed the activation of smaller atrial zones not reached by longer waves due to the presence of conduction blockade zones (51, 52).

Situations associated with oxidative stress generate an increase in intracellular calcium, by modifying type 2 ryanodine and intracellular calcium receptors, causing cellular calcium accumulation and facilitating the generation of triggered activity, cell hypertrophy, cell apoptosis, and fibrosis (36).

Atrial fibrillation results in both electrophysiological and autonomic remodeling of the atrium. Remodeling was characterized by increased atrial sympathetic innervation and heterogeneity of this innervation. This conclusion was confirmed by images of the atrial sympathetic nerve terminals of dogs by positron emission tomography, as well as by the increase in tissue norepinephrine content (53).

Obesity and obstructive sleep apnea syndrome cause autonomic remodeling by sympathetic activation, and this interferes with calcium metabolism, precipitating activity triggered mainly in muscle invaginations of pulmonary veins rich in innervation of the autonomic nervous system (36).

It is important to emphasize that structural remodeling, in general, occurs prior to the arrhythmic clinical presentation and some components are irreversible. Therefore, early treatment of clinical situations associated with atrial fibrillation is recommended, even before the electrical manifestation in tachyarrhythmia (37).

Progression from paroxysmal to persistent AF peaks in the first year, between 4 and 9% depending on the patient's treatment center; then there is a continuous growth, reaching 18–25% in 5 years of follow-up, a situation influenced by the clinical profile of the patient (8).

### Biomarkers

In recent years, the adoption of clinical tools with the objective of stratifying the risk of stroke, the most used being the CHA2DS2VASC scheme (age, sex, heart failure, or previous neurological vascular event parameters) (54). Although widely accepted by cardiology societies, its ability to predict the event is suboptimal [C statistic, 0.64; 95% confidence interval (CI), 0.58–0.70] (55).

In this context, biomarkers try to fill this gap by adding the advantage in trying to express the severity and duration of the disease or the individual response to the studied insult. In relation to AF, it could generate data with greater precision in relation to thrombogenicity and the propensity to develop it in the future (56). Some types of biomarkers have been described related to stress or myocardial injury (troponins and natriuretic peptides), coagulopathies (D-dimer, plasminogen activator inhibitor, tissue factor, and P-selectin), endothelial damage (thrombomodulin, E-selectin, and von Willebrand factor), inflammatory (C-reactive protein, interleukin-6, and tumor necrosis factor-alpha), fibrosis and extracellular matrix turnover (transforming growth factor-b, myeloperoxidase, and metallopeptidases and their inhibitor), or genetic factors (micro-RNA and single-nucleotide polymorphisms) (57).

A balance is needed between complex laboratory tests in relation to clinical tools that are easy and quick to use in the wards. Currently, there is a greater value in detecting and confirming the patient really at low risk of thromboembolic phenomena, so biomarkers with high negative predictive value may have a value in the future of daily clinical practice (57).

### Justifications for the Association of Atrial Myopathy and Stroke

It is well known that AF is related to atrial remodeling, only that these changes can occur with or without arrhythmia, so is it possible that myocardiopathy can cause stroke, even occasionally in the absence of AF? (43, 44, 58–63).

It was identified that episodes of AF with 6 min are related to an increased risk of stroke, except that atrial remodeling takes a few weeks for its complete institution, that is, another inconsistency in the direct role of AF and the neurological event appears (16, 64). This suggests that in this group of patients, atrial structural changes occur prior to the diagnosis of AF and the neurological event.

An important data in this context of cardioembolic stroke physiopathology is temporal dissociation. It was described based on data that demonstrated that cerebrovascular events and the presence of episodes of AF were not linear, that is, in many cases the robust temporal correlation between the two clinical events was lacking (35).

The *ASSERT* study of 2,580 patients with pacemaker and stroke demonstrated that only 8% of patients had a record of AF 30 days before the stroke and 16% had the first arrhythmic event detected only after clinical ictus (36). This suggests that arrhythmia may be a marker of atrial cardiomyopathy, excluding the previous concept that AF was the direct cause of cardioembolism (35, 36).

Contributing to the hypothesis, we have identified the inability of rhythm control in AF strategies in stroke prevention in clinical follow-up after maintenance at a steady pace sinus (36). It is interesting that, following this concept, the amount of atrial fibrosis, captured by magnetic resonance, in cases of cryptogenic stroke is similar to the cases related to manifest AF and greater than that of the cases associated with other etiologies (35).

### Prothrombotic Status

The entire cascade of events associated with cardiomyopathy and atrial remodeling can generate damage to the myocytes and also an expression of prothrombotic factors on the atrial endothelial surface associated with platelet activation and inflammatory cells amplifying the prothrombotic status, mainly in the left atrial appendage (35, 37).

**Figure 3:**
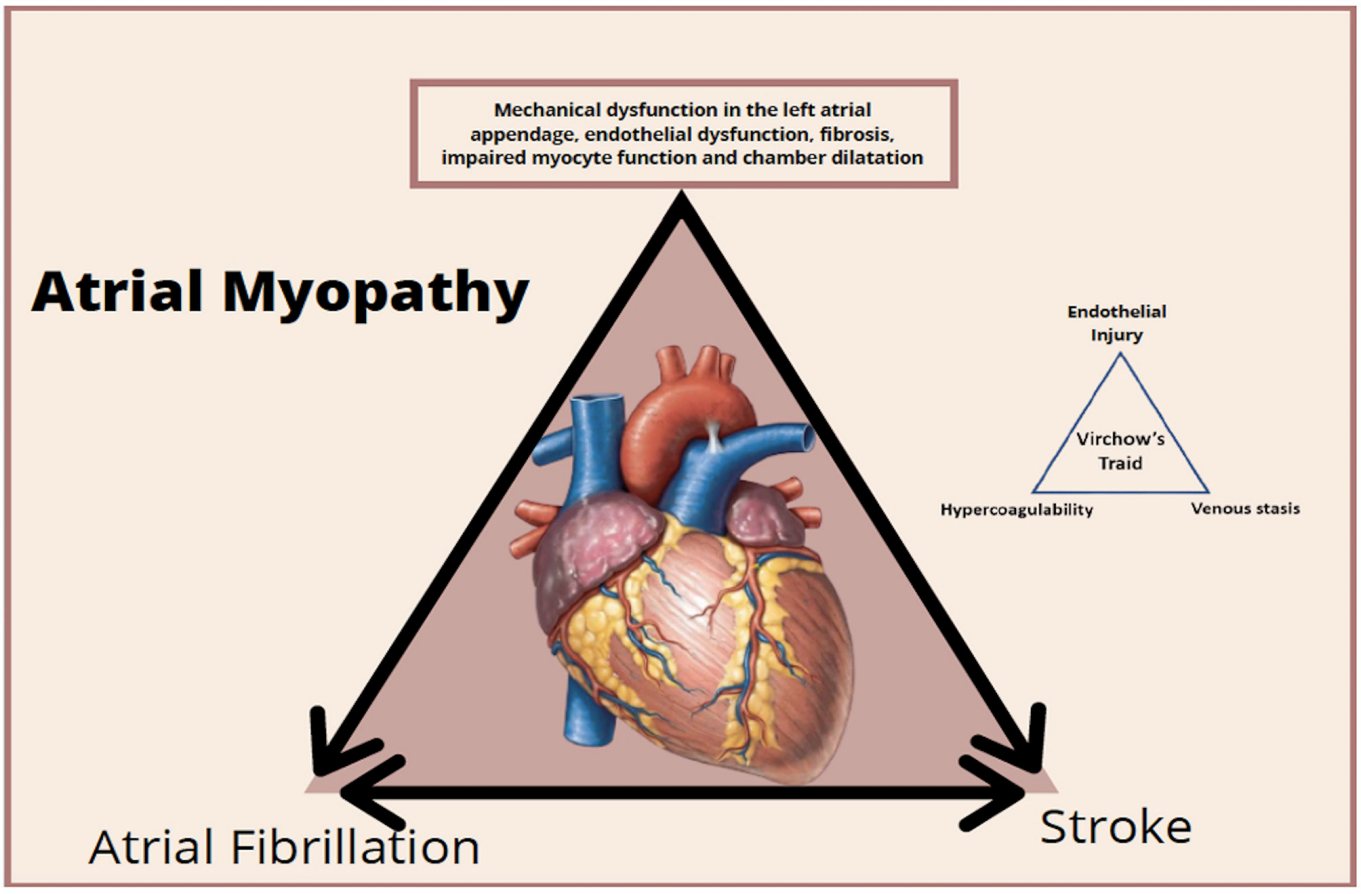
Clinical stages and prothrombotic status of atrial myopathy.

Situations and clinical signs associated with inflammation amplify the prothrombotic process because they are associated with the increased concentration of interleukin 6 and the activation of tissue factor, factor VII, and von Willebrand factor. This leads to endothelial damage, platelet activation, and greater sensitivity to thrombin, generating a vicious cycle between pro-thrombotic and inflammatory status (36) (Figure 3). It is important to clarify that atrial myopathy can present itself as AF initially but should always be considered thrombogenic in all the contexts of their clinical presentation (36). On the other hand, the burden of AF is important, since patients persistently and permanently have a higher stroke rate than found in paroxysmal; the arrhythmic load may be associated with a greater impairment atrial disease due to myopathy and, consequently, greater prothrombotic status (36).

What probably occurs in the presence of AF is that thrombogenic mechanisms are amplified through contractile dysfunction and blood stasis. This ends up meeting the results of clinical trials where a control of the rhythm of AF was not associated with reduced stroke, probably because it did not reduce the evolution of atrial heart disease (19).

## Clinical Implications

### Anticoagulation in Sinus Rhythm

Traditionally, the use of anticoagulants in patients with AF reduces the rate of stroke in the clinical follow-up by around 50–60% (65). In the *WARCEF* study, warfarin reduced the incidence of ischemic stroke in patients with HFREF compared to aspirin (HR 0.52, 95% CI 0.33–0.88, *p* = 0.0055) (66). Coumarins also reduce the chance of stroke in other types of heart disease, such as in patients with mechanical cardiac prostheses and with post-infarction ventricular thrombus (33). This pooling of evidence indirectly suggests that anticoagulation could prevent the risk of ischemic stroke in other cardiomyopathies (67).

The *WARSS* study was a multicenter randomized clinical trial that tested anticoagulation with warfarin versus aspirin in the secondary prevention of stroke in patients with a non-cardioembolic mechanism. Although the primary endpoint showed no benefit, p*ost-hoc* analysis showed that there was evidence of a benefit among those with NT-proBNP elevations above 750 pg/ml (HR 0.30, 95% CI 0.12–0.84; *p* = 0.021) (68). This constitutes a hypothesis that justified the performance of a clinical trial testing anticoagulation vs. antiplatelet therapy among patients with embolic stroke of undetermined source (ESUS) and atrial heart disease (67).

The *NAVIGATE ESUS* and *RE-SPECT ESUS* trials tested anticoagulation in ESUS, concluding that oral anticoagulation is not associated with reduced stroke recurrence rates compared to aspirin (69, 70). One of the reasons for the lack of benefit is the presence of overlapping potential embolic sources, characteristic of this population where more than 65% have more than one source. Certain sources (such as atrial heart disease, left ventricular disease, FOP, and cancer) are likely to benefit from anticoagulant therapy, whereas aortic plaques, cervical, or intracranial atherosclerosis may respond better to aspirin (71). Perhaps in the near future the ARCADIA trial will be able to answer this question; this study will include 1,100 patients with ESUS and with evidence of atrial heart disease in groups comparing apixaban and aspirin. The definition of heart disease will use at least one of the three selected biomarkers (PTFV1 >5,000 μV/ms on 12-lead ECG; Serum NT-proBNP >250 pg/ml; left atrial diameter index ≥3 cm/m^2^ on echocardiogram) (72). Although the optimal choice of biomarkers is not yet clear, the attempt to select patients with ESUS and heart disease is commendable in an attempt to demonstrate the role of anticoagulation in this subtype of stroke.

### Suggestion for Clinical Practice

Considering atrial heart disease (with or without the presence of AF) as a cause of embolic stroke, it could explain that one-third of strokes are considered cryptogenic (Figure 2) (73). Many are suspected of cardiomegaly clinically and through imaging, but only in one-third of patients an episode of AF is diagnosed even after 3 years of continuous cardiac monitoring (28). Therefore, the classification of cryptogenic may be overestimated, considering this association.

This sequence of facts implies the role of continuous monitoring to detect AF after an event considered initially cryptogenic. The absence of AF detection during the monitoring period cannot be the sole and exclusive reason for discontinuing anticoagulant therapy in association with other signs of atrial heart disease and the presence of clinical suspicion and stroke image (19).

As the institution of the concept of atrial heart disease is likely to control the concomitant risk factors that are associated with AF and atrial heart disease, they can be beneficial in reducing thromboembolic risk and not just restoring the atrial rhythm alone (74).

In the same way, sometimes AF can be considered as isolated, when it occurs in the absence of abnormal atrial substrate markers and without vascular risk factors, where the risk of stroke is minimized or even canceled (75).

## Conclusions

The traditional model exclusively associating the presence of AF in the genesis of thromboembolism is incomplete. The concept of atrial cardiopathy where cardioembolism occurs in a non-AF dependent manner fits better with current data. The future challenge is to effectively detect the various manifestations of atrial heart disease, generating direct implications for the identification of patients at risk of stroke and also for better management after a cardioembolic event.

## Author Contributions

All authors listed have made a substantial, direct and intellectual contribution to the work, and approved it for publication.

## Conflict of Interest

The authors declare that the research was conducted in the absence of any commercial or financial relationships that could be construed as a potential conflict of interest.

## Publisher's Note

All claims expressed in this article are solely those of the authors and do not necessarily represent those of their affiliated organizations, or those of the publisher, the editors and the reviewers. Any product that may be evaluated in this article, or claim that may be made by its manufacturer, is not guaranteed or endorsed by the publisher.
